# Editorial: Shared Genetic Risk Factors Among Psychiatric Diseases and Other Medical Diseases and Traits

**DOI:** 10.3389/fnins.2021.802064

**Published:** 2022-01-26

**Authors:** Adriana Lori, Fabio Coppedè, Silvia Pellegrini

**Affiliations:** ^1^Department of Psychiatry and Behavioral Sciences, Emory University, Atlanta, GA, United States; ^2^Department of Translational Research and of New Surgical and Medical Technologies, University of Pisa, Pisa, Italy; ^3^Department of Clinical and Experimental Medicine, University of Pisa, Pisa, Italy

**Keywords:** GWAS, PRS, EWAS, PTSD, ASD, CCM, Phonology, blood pressure

Genetic characterization of psychiatric disorders transcends diagnostic boundaries, suggesting substantial pleiotropy of contributing genes. Indeed, recent large-scale genome-wide association studies (GWAS) have highlighted both (a) the polygenic architecture of the majority of complex neuropsychiatric disorders [e.g., post-traumatic stress disorder (PTSD), schizophrenia (SCZ), major depressive disorder (MDD), autism spectrum disorders (ASD), attention deficit hyperactivity disorders (ADHD)] and (b) patterns of genetic overlap across different disorders.

The detection of these pleiotropic effects may facilitate the identification of individuals at risk of developing a mosaic of symptoms rather than a specific psychiatric disease. On the other hand, finding unique genetic signatures may help characterize distinct diagnostic categories. Identifying such effects will elucidate the underlying biology of psychiatric disorders, allowing for the development of targeted therapies.

The series of articles collected for this Research Topic reflects the current direction in psychiatry genetics and the recent efforts made by psychiatric research groups (e.g., Psychiatric Genomics Consortium -PGC; https://www.med.unc.edu/pgc/) to detect the nature and pleiotropic genetic mechanisms underlying these disorders.

The authors of these article's series have applied several variegated strategies to identify either pleiotropic or unique genes in psychiatric diseases: whole-genome sequencing to identify specific mutations in affected families (Han et al.); a candidate gene approach to identify variants associated with overlapping psychiatric diseases (Ma et al.) or to identify shared risk genes in overlapping traits (Unger et al.); analyses of different methylation (Bainomugisa et al.) or transcriptomic (Garrett et al.) patterns; the use of large genome-wide association study (GWAS) datasets from PGC to calculate polygenic risk scores (PRSs) associated with comorbid traits (Swart et al.; Sumner et al.). The PRS evaluates the genetic liability of several variants into a single per-individual score. PRS is calculated using two datasets: GWAS summary statistic from a training dataset and genome-wide data of a target population. For each set of variants, the PRS score is calculated as their sum weighted by their effect size for each subject in the target population. A significant association between the PRS and the trait in the target population establishes a common polygenic basis of the two (training and target) phenotypes.

In the first paper, Han et al. performed whole-exome sequencing in a large Chinese family diagnosed with Cerebral Cavernous Malformation (CCM), an autosomal dominant inherited disease with incomplete penetrance and heterogeneous symptoms, to identify possible causing mutations. These authors identified a novel missense variant (c.331G>C; pA111P; chr7:45104104) in the *CCM2* gene (NM_031443, NP_113631), never described before in public databases. Only family members carrying this variant showed MRI lesions that indicated a 100% neuroradiological penetrance. The pathogenetic role of this variant was predicted by *in silico* analysis of the protein structure, whose mutated forms showed a change of intramolecular hydrogen bonds causing alteration of the protein folding with the destruction of the phosphotyrosine binding domain. This study contributes to a better understanding of the spectrum of gene mutations in CCMs.

In the following two articles, the authors performed candidate gene analyses but with different approaches. Ma et al. used a traditional approach, exploring the potential contribution of *SHANK* genes to the genetic overlap between ADHD and ASD. The authors recruited children with ADHD, ASD, or ADHD plus ASD diagnosis (three clinical groups) and two control groups (community control group of typically developing boys and constructed matched pseudo-control from ADHD family) for genetic analyses of *SHANK2* and *SHANK3* genes, assessed using Tag-SNPs (single nucleotide polymorphisms). All subjects were of East Asian Ancestry. No differences in allele frequencies were observed among the three clinical groups or between the two control groups. Significant associations were observed between some *SHANK2* SNPs and aggregated groups of cases vs. aggregated controls, and between a combination of the different cases (e.g., ADHD and ADHD plus ASD) vs. aggregated controls. This study shows for the first time a pleiotropic effect of *SHANK2*, but not *SHANK3* variants, in ASD and ADHD in an East Asian population.

Unger et al. explored the shared genetic signatures of four phonological impairments (dyslexia, specific language impairment, dyscalculia, and logopenic variant of primary progressive aphasia), which are often associated with learning disorders, and in some cases, with late in life onset of neurodegenerative disease. These authors performed first a systematic literature review to identify overlapping genes associated with at least two of these conditions, then a gene ontology term enrichment analysis by using *learning* and *neurodevelopment* as main topics, and finally a gene expression analysis by using the Brain Expression Atlas in two key brain regions of the language network (Broca and Wernicke), whose functional connectivity improves linguistic performance. Twelve genes were overexpressed in both language areas, and three of them showed differential activation in the Wernicke (*DNAAF4, FOXP2*) or the Broca (*ATP2C2*) region, suggesting specific regional expression and functional specialization. In summary, a combination of literature mining, gene ontology term enrichment analysis, and brain gene expression analysis was used to identify candidate genes underlying a selected group of disorders.

The last four papers focused on identifying genes associated with PTSD and other comorbid psychiatric diseases (e.g., depression) or co-occurring conditions (e.g., coronary heart diseases, cardiovascular disorders, metabolic syndromes, and migraine headaches).

Swart et al. carried out the first GWAS of PTSD in an African population. Several genetic loci showed nominally significant association with PTSD (*PARK2, CUB, CSMD1, DOCK4, ABCA8*, and *C3orf8*), although none of them reached a genome-wide significance. Some of these genes were previously identified by other PTSD-GWAS on civilian and military populations (e.g., *PARK2, CUB*, and *CSMD1*; Nievergelt et al., 2015), or in association with other psychiatric diseases (e.g., *DOCK4* in *SCZ* and ASD; Schizophrenia Psychiatric Genome-Wide Association Study Consortium, 2011; Stephan et al., 2012; Woo et al., 2017; Koomar and Michaelson, 2020), and other neurodevelopmental disorders (e.g., *PARK2* in Parkinson's Disease; Jarick et al., 2014). These authors also observed a significant cross ancestry (European–African) correlation between PGC-PTSD polygenic risk scores and PTSD status, PTSD symptom severity, and metabolic symptoms.

Sumner et al. used the genomic data from the PGC-PTSD Consortium and PGC-PTSD Physical Health Working Group, including 11 studies of 72,224 European and African ancestry individuals, to explore the contributions of PTSD symptoms to blood pressure outcomes. A blood pressure PRS was used to determine whether it moderated the associations of PTSD with systolic and diastolic blood pressure. PTSD symptoms have been previously associated with systolic blood pressure (SBP) and diastolic blood pressure (DBP) levels but in heterogeneous ways and with small effect sizes. Although underlying polygenic scores significantly predicted blood pressure levels, this genetic metric did not modify the associations of PTSD symptoms with SBP or DBP. The results of this study created the ground for further research to better understand the correlation between PTSD, blood pressure, and cardiovascular risks.

Bainomugisa et al. investigated the comorbidity relationship between PTSD and migraine in monozygotic twins of European ancestry. These authors first assessed differences in methylation patterns by performing an epigenome-wide association study (EWAS) in six pairs of twins discordant for PTSD. They validated 11 candidate genes from previous PTSD studies (e.g., *DOCK2, DICER1*, and *ADCYAP1*) and identified seven novel CpGs associated with this disorder. Next, Bainomugisa et al. compared EWAS signals from a second independent set of twins discordant for migraine symptoms, with the 10% FDR significant CpGs identified in the PTSD-twin analysis. *DAPK2* and *TM6SF2* were identified as top, overlapping genes between PTSD and migraine. AMPK and longevity regulating pathways were overrepresented in genes associated with both PTSD and migraine. Despite the small sample size, the authors could replicate previous PTSD findings and identify possible overlapping mechanisms between PTSD and migraine.

Finally, Garrett et al. investigated the pathophysiology of PTSD by analyzing blood differential gene expression in three separated trauma-exposed cohorts, and then performed a meta-analysis. Their work represents the largest PTSD transcriptome study performed to date. The results of this study suggest a role for differential expression of inflammatory genes and pathways in PTSD, including *IL1B* and a new possible candidate gene, *ILK*. Interestingly, *ILK* gene has been associated with fetal alcohol spectrum disorder and Alzheimer's disease in animal models. Gene weight co-expression analyses in one non-Hispanic White cohort (MIRECC/Duke study) identified several modules associated with PTSD, two of which were also associated with MDD, assessed by either SCID (Structured Clinical Interview for Diagnostic) statistical manual disease (First et al., 1994), a gold standard interview for MDD, PHQ-9 (Patient Health Questionnaire 9; Kroenke et al., 2001), or the Mini-International Neuropsychiatry Interview (Sheehan et al., 1998). These data suggest the existence of common biological pathways underlying PTSD and MDD.

To conclude, this Research Topic—collection of articles—reports evidence of novel genes and pathways with pleiotropic effects in several psychiatric disorders, including ADHD, ASD, PTSD, MDD, as well as in migraine and phonological impairments.

Of note, three papers from this collection focused on non-European populations: Han et al. on a Chinese family; Swart et al. on an African Cohort; Ma et al. on East Asian boys; a fourth paper performed a transcriptome meta-analysis in a multiethnic population (Garrett et al.), and a fifth one calculated PRSs from both European and African Ancestry cohorts (Sumner et al.). As the vast majority of the genetic studies published to-date have taken into consideration only individuals of European ancestry, these five papers carry important implications that may allow the extension of the understanding of the biological mechanisms underlying psychiatric diseases from Europeans to other populations.

## Author Contributions

All authors wrote and equally contributed to the article and approved the submitted version.

## Conflict of Interest

The authors declare that the research was conducted in the absence of any commercial or financial relationships that could be construed as a potential conflict of interest.

## Publisher's Note

All claims expressed in this article are solely those of the authors and do not necessarily represent those of their affiliated organizations, or those of the publisher, the editors and the reviewers. Any product that may be evaluated in this article, or claim that may be made by its manufacturer, is not guaranteed or endorsed by the publisher.

## References

[B1] First M. B. Frances A. J. Pincus H. A. Vettorello N. Davis W. W. (1994). DSM-IV in progress. Changes in substance-related, schizophrenic, and other primarily adult disorders. Hosp. Commun. Psychiatry 45, 18–20. 10.1176/ps.45.1.18 8125454

[B2] Jarick I. Volckmar A. L. Putter C. Pechlivanis S. Nguyen T. T. Dauvermann M. R.. (2014). Genome-wide analysis of rare copy number variations reveals PARK2 as a candidate gene for attention-deficit/hyperactivity disorder. Mol. Psychiatry 19, 115–121. 10.1038/mp.2012.161 23164820 PMC3873032

[B3] Koomar T. Michaelson J. J. (2020). Genetic intersections of language and neuropsychiatric conditions. Curr. Psychiatry Rep. 22:4. 10.1007/s11920-019-1123-z 31953567

[B4] Kroenke K. Spitzer R. L. Williams J. B. (2001). The PHQ-9: validity of a brief depression severity measure. J. Gen. Intern. Med. 16, 606–613. 10.1046/j.1525-1497.2001.016009606.x 11556941 PMC1495268

[B5] Nievergelt C. M. Maihofer A. X. Mustapic M. Yurgil K. A. Schork N. J. Miller M. W.. (2015). Genomic predictors of combat stress vulnerability and resilience in U.S. marines: a genome-wide association study across multiple ancestries implicates PRTFDC1 as a potential PTSD gene. Psychoneuroendocrinology 51, 459–471. 10.1016/j.psyneuen.2014.10.017 25456346

[B6] Schizophrenia Psychiatric Genome-Wide Association Study Consortium (2011). Genome-wide association study identifies five new schizophrenia loci. Nat. Genet. 43, 969–976. 10.1038/ng.940 21926974 PMC3303194

[B7] Sheehan D. V. Lecrubier Y. Sheehan K. H. Amorim P. Janavs J. Weiller E.. (1998). The Mini-International Neuropsychiatric Interview (M.I.N.I.): the development and validation of a structured diagnostic psychiatric interview for DSM-IV and ICD-10. J. Clin. Psychiatry 59(Suppl. 20), 22–33;quiz: 34–57. 9881538

[B8] Stephan A. H. Barres B. A. Stevens B. (2012). The complement system: an unexpected role in synaptic pruning during development and disease. Annu. Rev. Neurosci. 35, 369–389. 10.1146/annurev-neuro-061010-113810 22715882

[B9] Woo H. J. Yu C. Kumar K. Reifman J. (2017). Large-scale interaction effects reveal missing heritability in schizophrenia, bipolar disorder and posttraumatic stress disorder. Transl. Psychiatry 7:e1089. 10.1038/tp.2017.61 28398343 PMC5416702

